# Diagnostic Value of Native T1 and T2 Mapping in Differentiating Clinically Suspected Amyloidosis and Hypertrophic Cardiomyopathy

**DOI:** 10.3390/diagnostics16101558

**Published:** 2026-05-20

**Authors:** Sena Unal, Caglar Uzun, Sena Bozer Uludag, Cuneyt Yamak, Turkan Seda Tan, Elif Peker

**Affiliations:** 1 Department of Radiology, School of Medicine, Ankara University, Hacettepe Mahallesi A. Adnan Saygun Cad. No: 35, 06230 Altındağ, Ankara, Türkiye; drsenaunal@gmail.com (S.U.); cuneytyamak@gmail.com (C.Y.); elifozyurek0@yahoo.com (E.P.); 2 Department of Radiology, Tosya State Hospital, A. Raşit Urkaya Sok., Dilküşah Mahallesi No: 2, 37300 Tosya, Kastamonu, Türkiye; senabozer@gmail.com; 3 Department of Cardiology, School of Medicine, Ankara University, Hacettepe Mahallesi A. Adnan Saygun Cad. No: 35, 06230 Altındağ, Ankara, Türkiye; skurklu@ankara.edu.tr

**Keywords:** amyloidosis, hypertrophic cardiomyopathy, cardiac MRI, T1 mapping, T2 mapping

## Abstract

**Background/Objectives:** Differentiating clinically suspected cardiac amyloidosis from hypertrophic cardiomyopathy (HCM) remains a significant clinical challenge, especially when contrast-enhanced imaging is contraindicated. This study evaluated the potential diagnostic utility of non-contrast cardiac MRI parameters, specifically native T1 and T2 mapping, as supportive indicators in this differential diagnosis. **Methods:** This retrospective single-center study included 20 patients with clinically suspected amyloidosis (based on combined clinical and echocardiographic assessment), 20 patients with HCM, and 20 healthy controls. Cine imaging and native T1/T2 mapping were analyzed. Myocardial, blood-pool, and liver T1/T2 values, along with morphological parameters, were recorded. N-terminal pro–B-type natriuretic peptide (NT-proBNP) and troponin levels, when available, were documented retrospectively for descriptive purposes. Receiver operating characteristic (ROC) analyses were performed to assess the discriminatory performance of imaging parameters. **Results:** Patients in the suspected amyloidosis group demonstrated significantly higher myocardial, blood-pool, and liver T1 values, as well as higher myocardial T2 values, compared with both the HCM and control groups (*p* < 0.001). Myocardial T1 showed strong discriminatory performance for differentiating suspected amyloidosis from controls (cut-off 1061 ms, AUC = 0.975). In distinguishing suspected amyloidosis from HCM, blood-pool T1 (AUC = 0.900) and myocardial T1 (AUC = 0.938) provided the highest diagnostic performance. Additionally, elevated NT-proBNP (>1000 pg/mL in 93% of tested cases) and troponin levels were observed in the suspected amyloidosis group, consistent with increased myocardial stress. **Conclusions:** Native T1 and T2 mapping may offer valuable supportive information in differentiating clinically suspected amyloidosis from HCM on non-contrast MRI. Myocardial and blood-pool T1 values appear to provide complementary tissue characterization, which may be particularly useful when gadolinium administration or invasive procedures are not feasible. These findings suggest a role for non-contrast mapping in the diagnostic workup but require further validation in larger, biopsy-confirmed multicenter cohorts.

## 1. Introduction

Cardiac amyloidosis results from the infiltration of the myocardium by amyloid fibrils. Deposition of these fibrils leads to myocardial thickening and often manifests as left ventricular hypertrophy, which can be challenging to distinguish from hypertrophic cardiomyopathy (HCM) in clinical practice [1]. In contrast to amyloidosis, HCM is caused by genetic mutations in the cardiac sarcomere and is not a systemic or infiltrative disease [2]. Cardiac amyloidosis represents a particularly important diagnostic challenge, as it may act as a phenocopy of HCM despite fundamentally different underlying mechanisms and therapeutic implications [3]. Although both conditions cause left ventricular hypertrophy, they differ significantly in treatment and prognosis, making an accurate differentiation essential [1].

Various diagnostic tools are utilized for the evaluation of cardiac amyloidosis. Electrocardiography and echocardiography are widely used first-line tools that may raise suspicion for the disease. However, cardiac magnetic resonance imaging (MRI) is a superior modality for assessing cardiac function and tissue characteristics [4]. Typical contrast enhancement patterns on cardiac MRI for both cardiac amyloidosis and HCM can be highly suggestive. Previous studies have focused on differentiating these entities using contrast-enhanced patterns and morphological features [5,6].

However, contrast administration may not be possible in patients with renal dysfunction, who represent a significant portion of the amyloidosis population. Furthermore, the gold-standard diagnostic procedures, such as endomyocardial biopsy or specialized scintigraphy, may not always be feasible or accessible in all clinical settings. Therefore, identifying distinguishing features on non-contrast MRI is of great clinical value for the management of these patients.

The aim of this study was to identify non-contrast MRI parameters, such as native T1 and T2 mapping, that may contribute to the differentiation of clinically suspected amyloidosis from HCM, providing supportive evidence when contrast-enhanced imaging or invasive gold-standard methods are limited.

## 2. Materials and Methods

Patient Selection and Clinical Phenotyping

The institutional ethics committee approved this retrospective single-center study. We evaluated a total of 62 patients referred for cardiac MRI to investigate left ventricular hypertrophy (LVH) between 2016 and 2021.

Clinically Suspected Amyloidosis Group:

The clinically suspected amyloidosis cohort (*n* = 20) was defined based on a retrospective multidisciplinary clinical assessment reflecting real-world diagnostic practice. Patients were classified based on a combined evaluation of echocardiographic findings and clinical context, both of which were required to support the suspected phenotype.

Echocardiographic reports were systematically reviewed for characteristic features of infiltrative disease, including increased myocardial echogenicity (“speckled” appearance), biatrial enlargement, restrictive filling physiology, or an apical sparing pattern on longitudinal strain analysis. The presence of one or more of these findings, in an appropriate clinical context, supported inclusion in the suspected amyloidosis group.

Clinical context was defined by the presence of findings suggestive of an infiltrative or systemic process, including unexplained left ventricular hypertrophy, heart failure symptoms, and/or extracardiac manifestations such as renal function impairment or extracardiac tissue biopsy. Regarding systemic involvement, extracardiac tissue biopsy results (e.g., fat pad, skin, or renal biopsy) were documented for 15 patients; 6 patients had positive results for amyloid deposition, while 9 were negative. In the remaining 5 patients, no biopsy was performed.

We acknowledge that this multimodal, retrospective definition may introduce incorporation bias, as some of the clinical features used for group assignment may overlap with the myocardial alterations assessed by native T1 and T2 mapping.

Biochemical Profile: Cardiac biomarkers (NT-proBNP and troponin) were not used as inclusion criteria. These parameters were evaluated retrospectively after cohort assignment and are presented solely to describe the clinical phenotype. When available, elevated values were interpreted as nonspecific indicators of myocardial stress and chronic injury rather than diagnostic evidence of amyloidosis. In this cohort, 75% (*n* = 15) had NT-proBNP data and 80% (*n* = 16) had troponin levels available. Notably, 93.3% of the tested patients exhibited NT-proBNP levels > 1000 pg/mL (institutional normal reference range: 0–125 pg/mL), with values ranging from 836.8 pg/mL to over 35,000 pg/mL, and 81.2% showed elevated troponin levels (ranging from 15.58 ng/mL to 423.8 ng/mL, institutional normal reference range: 0–14 ng/mL), providing strong biochemical evidence of myocardial stress and injury. These values should be interpreted as nonspecific markers of myocardial stress and chronic injury rather than diagnostic evidence, and are reported solely for descriptive purposes after cohort definition.

Hypertrophic Cardiomyopathy (HCM) Group:

Hypertrophic cardiomyopathy was defined according to current guideline-based criteria [7], defined as left ventricular wall thickness ≥15 mm in one or more myocardial segments without potential causes of secondary myocardial hypertrophy. Clinical records, echocardiographic findings, and cardiac MRI were reviewed to exclude secondary causes and phenocopies. Family history was assessed when available to support the diagnosis of primary HCM. Genetic testing was not systematically available due to the retrospective design. Furthermore, patients in the HCM group were specifically screened to confirm they did not meet any of the clinical or biochemical criteria (such as markedly elevated NT-proBNP or extra-cardiac biopsy suspicion) used for the suspected amyloidosis group. Twenty patients fulfilling these criteria were included in the HCM group.

Control Group:

The control group consisted of 20 individuals who underwent cardiac MRI for mild or nonspecific symptoms but demonstrated no morphological, functional, or tissue abnormalities on MRI, serving as a baseline for normal mapping values.

Inclusion and Exclusion Criteria

Inclusion criteria were (i) age > 18 years, (ii) a multidisciplinary clinical suspicion of cardiac amyloidosis or imaging findings consistent with primary HCM, and (iii) availability of native T1 and T2 mapping data.

Exclusion criteria were defined as follows: (i) non-diagnostic image quality (*n* = 3), (ii) failure to meet the standardized imaging criteria for HCM (*n* = 7), or (iii) inconclusive clinical or biochemical markers that did not meet the predefined threshold for suspected cardiac amyloidosis (*n* = 12).

After applying these criteria, the final study cohort consisted of 40 patients (20 suspected amyloidosis and 20 HCM). For the control group (*n* = 20), normality was confirmed not only by normal MRI findings but also through a comprehensive review of medical records and ECGs to ensure the absence of any known cardiovascular or systemic disease.

MRI Acquisition and Image Analysis

All MR examinations were performed on a 1.5 T scanner (Aera, Siemens Healthcare, Erlangen, Germany) with use of an 18-channel body coil, in accordance with the recommendations of the Society for Cardiovascular Magnetic Resonance and the Working Group of the European Society of Cardiology [6].

For routine cardiac MRIs, 2-chamber, 3-chamber, and 4-chamber cine images, as well as left ventricular short-axis cine images, were obtained. Cine images were acquired using a balanced steady-state free precession technique (TR/TE, 42.98/1.33; flip angle, 80°; and spatial resolution, 1.63 × 1.63 × 8 mm) with parallel imaging (generalized autocalibrating partial parallel acquisition [GRAPPA] factor 3) with breath-holding in expiration and retrospective gating. Additionally, native T1 mapping images were acquired using a heart-rate-corrected 5(3)3 protocol with a modified Look-Locker inversion recovery (MOLLI) sequence, and T2 mapping images were acquired using a TrueFISP single-shot readout. T1 and T2 mapping were acquired using standardized acquisition parameters. For T1 mapping, the sequence parameters included a repetition time (TR) of 272 ms, an echo time (TE) of 1.12 ms, a field of view (FOV) of 360 × 360 mm, a slice thickness of 8 mm, and three slices. For T2 mapping, the acquisition was performed with a TR of 188.29 ms, a TE of 1.06 ms, an FOV of 360 × 360 mm, a slice thickness of 8 mm, and three slices.

**Figure 1:**
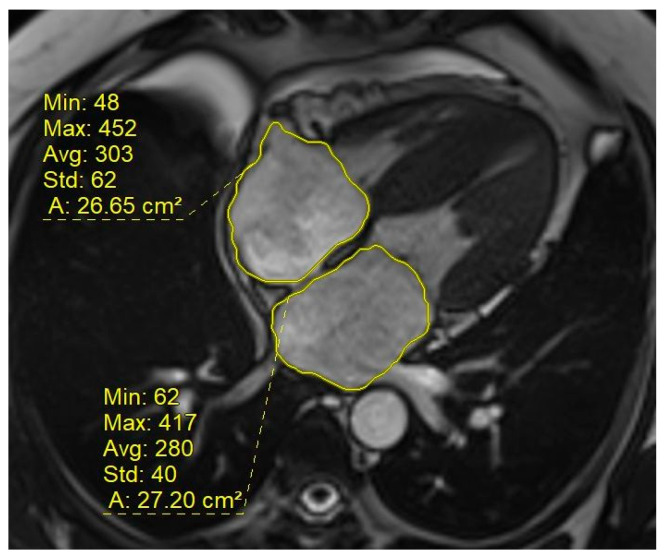
Measurement of atrial areas from four-chamber cine images.

**Figure 2:**
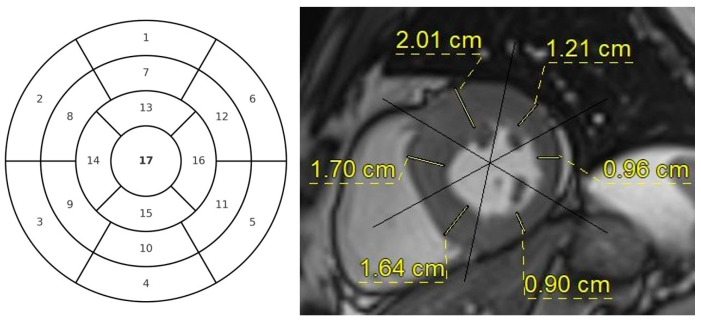
Segmentation of the left ventricle and measurement of end-diastolic myocardial thickness from cine short-axis images.

Morphological and functional assessment was performed via cine images. From the 4-chamber images, the areas of both atria were measured at end-ventricular systole and classified as normal or enlarged (>24 cm^2^) (Figure 1). The left ventricle was divided into segments on cine short-axis images, and end-diastolic myocardial thickness was measured and documented for 16 segments (Figure 2). A myocardial thickness of 15 mm or greater was considered increased. Left ventricle end-systolic and end-diastolic volumes were measured from short-axis images using the imaging software workstation (syngo.via VB30A HF06, Siemens Healthineers, Erlangen, Germany). Ejection fraction (EF) values and end-diastolic volume indices (EDVi) were calculated.

**Figure 3:**
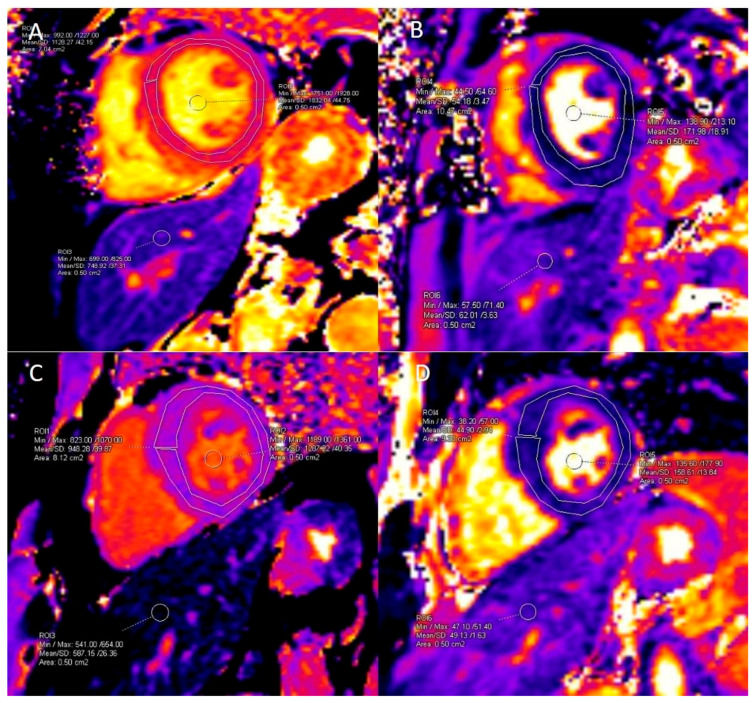
T1 and T2 mapping measurements from the left ventricular myocardium, blood-pool, and liver in an amyloidosis (**A**,**B**) and an HCM (**C**,**D**) patient. Institutional reference values for myocardium: T1 = 1001 ± 25 ms, T2 = 46 ± 2.8 ms.

T1 and T2 mapping measurements were performed from the left ventricular myocardium covering the entire myocardium by using free-hand ROIs and by placing 5 cm^2^ ROIs in the blood-pool and in the non-vascular portion of the liver parenchyma using the imaging software workstation (Figure 3). At our institution, center-specific normal reference values for native mapping were established using the same scanner and acquisition protocol. The reference myocardial native T1 value is 1001 ± 25 ms and the reference myocardial T2 value is 46 ± 2.8 ms.

T1 and T2 values, myocardial thicknesses, EF values, EDVi, and atrial sizes were compared between the suspected amyloidosis and HCM groups, as well as between each group and the control group.

During the morphological evaluation, the presence of pericardial and pleural effusions was also noted. Pericardial fluid was assessed for its distribution and relative thickness, and any associated pleural effusions were documented from the available cine sequences. These findings were integrated into the overall clinical and morphological characterization of the patients to provide a more comprehensive overview of each cohort.

Late gadolinium enhancement (LGE) was not included in the primary analysis to reflect real-world clinical constraints. In our cohort, 16 of 20 patients (80%) with suspected amyloidosis underwent non-contrast MRI due to renal dysfunction, resulting in insufficient LGE data for a reliable and unbiased comparison between the groups.

Biochemical Markers

Electronic medical records were reviewed for serum NT-proBNP and troponin levels to complement the imaging findings. In the suspected amyloidosis group, NT-proBNP data were available for 15 patients (75%), while troponin levels were available for 16 patients (80%). To ensure clinical relevance, measurements obtained within one year of the MRI exam were included, with the vast majority (*n* = 13 for both markers) performed within a narrow window of less than 1.5 months from the imaging study, providing a strong temporal correlation. Given the chronic and progressive nature of amyloidosis, these values were considered representative of the patients’ baseline status at the time of MRI. In contrast, these markers were not routinely available for the HCM group due to their generally stable clinical status. Consequently, a formal statistical comparison between the groups regarding laboratory values was not performed; instead, these markers were used primarily to support the clinical phenotyping of the suspected amyloidosis cohort where data were available.

Reproducibility Analysis

To evaluate the reproducibility of the native T1 and T2 mapping measurements, inter-observer and intra-observer variability analyses were performed on a randomly selected subset of 15 subjects. For intra-observer reliability, the primary radiologist (S.U.) re-evaluated the images more than one year after the initial assessment, ensuring complete blinding to the original data and eliminating any potential recall bias. For inter-observer reliability, a second radiologist (E.P.) with 14 years of experience in cardiac imaging, blinded to the initial findings and clinical data, independently performed the ROI measurements using the same standardized protocol. Reliability was assessed using the intraclass correlation coefficient (ICC) with a two-way mixed-effects model and absolute agreement type. ICC values were interpreted as follows: <0.50 (poor), 0.50–0.75 (moderate), 0.75–0.90 (good), and >0.90 (excellent).

Statistical Analysis

Statistical analyses were performed using SPSS software (version 15.0; SPSS Inc., Chicago, IL, USA). Descriptive statistics were expressed as mean ± standard deviation for metric variables, median (minimum–maximum) for non-parametrically distributed variables, and frequency (percentage) for categorical variables. The distribution of the data was assessed using the Shapiro–Wilk test. For comparisons between two independent groups, Student’s *t*-test was applied when parametric test assumptions were met; otherwise, the Mann–Whitney *U* test was used. Categorical variables were compared using the Chi-square or Fisher’s exact test. Receiver operating characteristic (ROC) curve analysis was performed to determine the area under the curve (AUC), optimal cut-off values, sensitivity, specificity, and 95% confidence intervals. A *p*-value of <0.05 was considered statistically significant.

## 3. Results

The mean age was 59 ± 14 years for patients with clinically suspected amyloidosis (6 females, 14 males), 50 ± 13 years for patients with HCM (5 females, 15 males), and 32.6 ± 9.8 years for the control group (9 females, 11 males).

### 3.1. Group Comparisons

#### 3.1.1. Clinically Suspected Amyloidosis vs. Control Group

**Figure 4:**
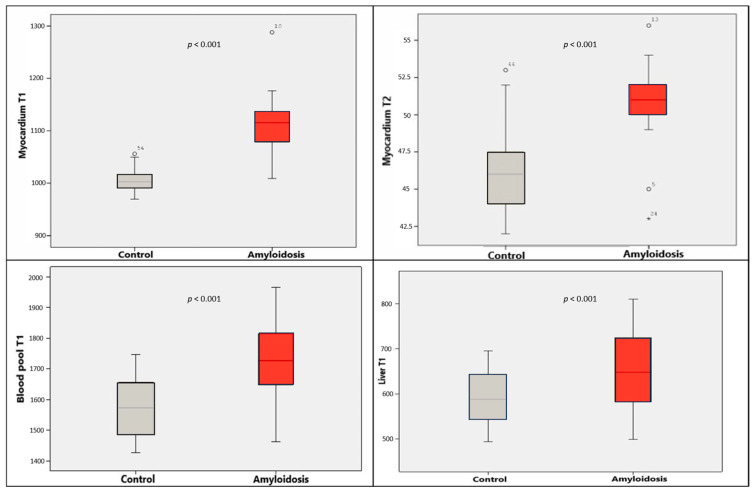
Comparison of myocardial, blood-pool, and liver T1 values and myocardial T2 values between the control and amyloidosis groups. Gray boxes represent the control group, and red boxes represent the suspected amyloidosis group.

Native T1 values for the myocardium, liver, and blood-pool were significantly higher in the amyloidosis group compared to the control group (*p* < 0.001). While T2 values for the liver and blood-pool showed no significant difference, myocardial T2 values were significantly elevated in the amyloidosis group (Table 1) (Figure 4).

ROC analysis indicated that a myocardial T1 cut-off of 1061 ms provided the highest discriminatory performance for identifying amyloidosis, with an AUC of 0.975 (*p* < 0.001), sensitivity of 85%, specificity of 100%, and an overall accuracy of 92.5%. A blood-pool T1 cut-off of 1627.5 ms also showed significant performance (AUC = 0.836, *p* < 0.001), albeit with lower specificity (65%). In contrast, liver T1 values (cut-off 622 ms) did not reach statistical significance (*p* = 0.23). For myocardial T2 mapping, a cut-off of 48.5 ms yielded an AUC of 0.816 (*p* = 0.001), with high sensitivity (95%) and acceptable specificity (80%) (Table 2).

**Figure 5:**
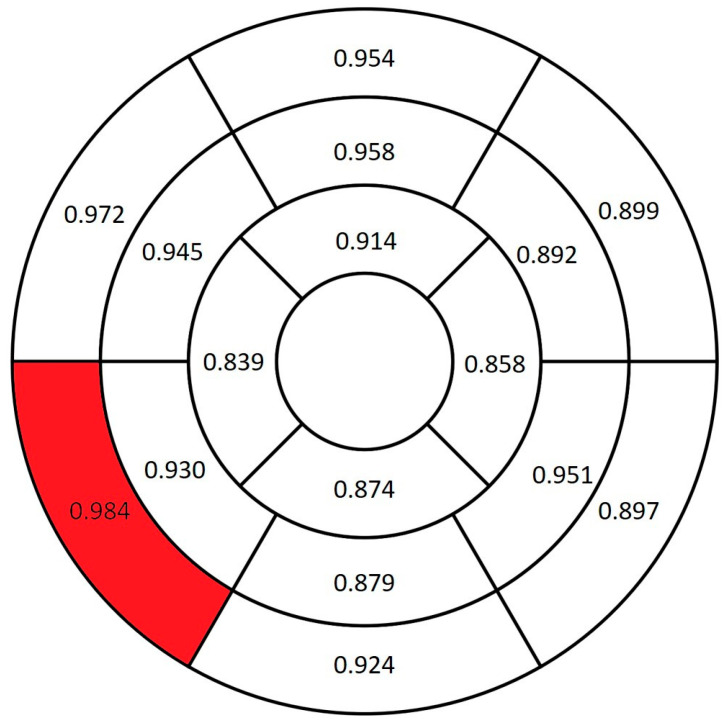
AUC values of myocardial thickness for differentiating between the amyloidosis and control groups. The red-highlighted segment indicates the highest AUC value.

The amyloidosis group had significantly greater myocardial thickness across all segments compared with the control group (*p* < 0.001). The basal inferoseptal segment had the greatest AUC value (0.984) (Figure 5).

#### 3.1.2. HCM vs. Control Group

**Figure 6:**
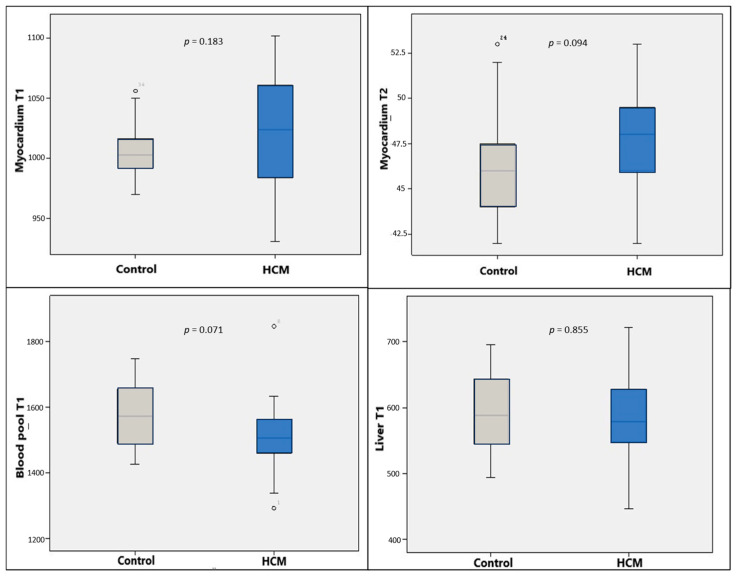
Comparison of myocardial, blood-pool, and liver T1 values and myocardial T2 values between the control and HCM groups. Gray boxes represent the control group, and blue boxes represent the HCM group.

When HCM patients were compared with the control group, no statistically significant differences were found in T1 and T2 values of the myocardium, blood-pool, and liver (*p* > 0.05) (Table 1) (Figure 6).

When myocardial thickness was compared, it was significantly greater in the HCM group across all segments (*p* < 0.001).

#### 3.1.3. Clinically Suspected Amyloidosis vs. HCM

**Figure 7:**
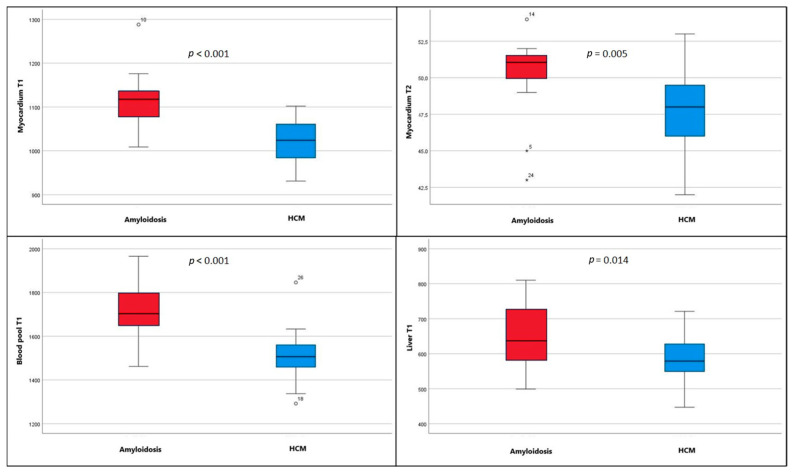
Comparison of myocardial, blood-pool, and liver T1 values and myocardial T2 values between the amyloidosis and HCM groups. Red boxes represent the suspected amyloidosis group, and blue boxes represent the HCM group.

Patients with suspected amyloidosis demonstrated significantly higher T1 values (myocardium, liver, and blood-pool) and significantly higher myocardial T2 values compared to HCM patients (Table 1, Figure 7).

**Figure 8:**
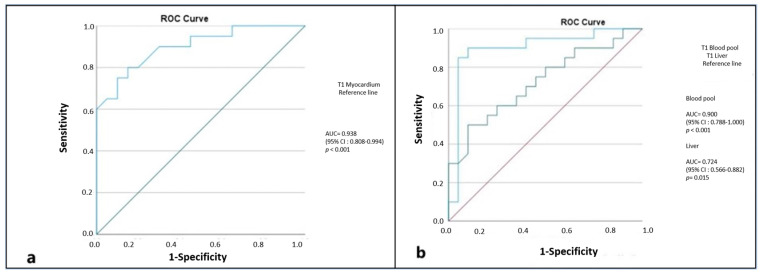
ROC analysis of myocardium (**a**), blood-pool, and liver (**b**) for differentiating amyloidosis from HCM.

In distinguishing between the suspected amyloidosis and HCM groups, myocardial T1 (AUC = 0.938, cut-off 1071 ms) and blood-pool T1 (AUC = 0.900, cut-off 1597 ms) provided the highest discriminatory value, whereas liver T1 showed lower performance (Table 3) (Figure 8).

Regarding functional and morphological parameters, EF values were significantly higher in HCM group compared to the amyloidosis group. No significant differences were observed in EDVi or atrial size (Table 4).

**Figure 9:**
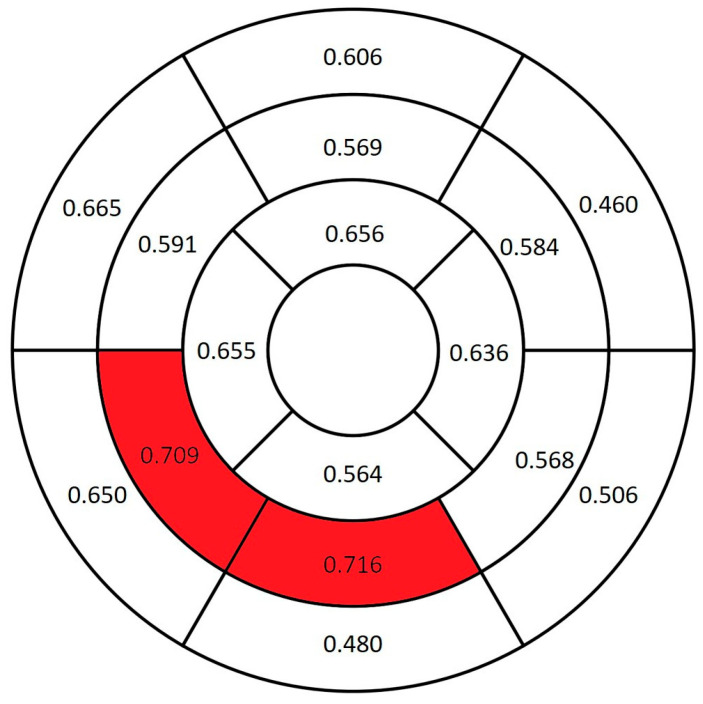
AUC values of myocardial thickness for differentiating between the amyloidosis and HCM groups. The red-highlighted segments indicate the highest AUC values.

Wall thickness in the mid-inferior segment (segment 10) was significantly greater in HCM patients (mean: 12.4 ± 5.1 mm, median: 12 mm) compared with suspected amyloidosis patients (mean: 9.4 ± 2.4 mm, median: 9 mm; *p* = 0.018). Similarly, in the mid-inferoseptal segment (segment 9), wall thickness was significantly higher in HCM group (mean: 16.3 ± 6.3 mm, median: 15 mm) than in amyloidosis group (mean: 12.5 ± 3.7 mm, median: 11.5 mm; *p* = 0.023). No significant differences were detected in the other segments (Figure 9).

Comparison of the frequency of segments with increased myocardial thickness (≥15 mm) revealed no significant differences between the amyloidosis and HCM groups in any segment (*p* > 0.05). In HCM group, increased thickness was most commonly observed in the mid-inferoseptal segment (55%), whereas in amyloidosis group, it was most frequently seen in the mid-inferoseptal and basal anterior segments (25% each).

The reproducibility analysis demonstrated high reliability for myocardial mapping measurements. Intra- and inter-observer intraclass correlation coefficients (ICC) for myocardial native T1 and T2 were 0.973 (95% CI: 0.936–0.990) and 0.877 (95% CI: 0.725–0.954), respectively. Blood-pool and liver mapping values also showed good to excellent reproducibility, with ICCs ranging from 0.667 to 0.968. These results confirm the robustness and consistency of the manual ROI-based measurement technique utilized in our protocol.

Extracardiac findings also showed notable differences between the groups. Pleural effusion was markedly more prevalent in the suspected amyloidosis group (13/20) compared to the HCM group (1/20). Only one patient in the HCM group exhibited a small amount of pleural effusion, whereas it was a common and more significant finding in the suspected amyloidosis group. Regarding pericardial involvement, although fluid was detected in both cohorts, it was mostly minimal in the HCM group. In contrast, patients with suspected amyloidosis often presented with more prominent pericardial effusions.

Laboratory data for the suspected amyloidosis group revealed a biochemical profile highly suggestive of significant myocardial stress and injury. Of the 15 patients with available NT-proBNP measurements, all 15 (100%) exhibited levels well above the institutional reference range (0–125 pg/mL). Notably, 14 out of these 15 patients (93.3%) had NT-proBNP values exceeding 1000 pg/mL, while the remaining patient had a value of >800 pg/mL. Regarding troponin levels (reference range: 0–14 ng/L), data were available for 16 patients, of whom 13 (81.2%) showed elevated levels, consistent with chronic myocardial injury. In contrast, such biochemical evaluations were not routinely performed for the HCM group as detailed in the Section 2.

## 4. Discussion

In the present study, we evaluated the potential of non-contrast MRI parameters to differentiate between patients with clinically suspected cardiac amyloidosis and those with HCM. Our findings suggest that native T1 and T2 mapping may serve as valuable supportive tools in this challenging differential diagnosis, particularly when gadolinium-based contrast agents are contraindicated. Within our cohort, patients in the suspected amyloidosis group exhibited significantly higher myocardial native T1 and T2 values compared to both HCM patients and healthy controls. While specific native T1 thresholds were identified for our center’s scanner and protocol, these values should be interpreted as illustrative of the marked tissue characterization differences between the two phenotypes rather than absolute diagnostic cut-offs. Notably, while elevated mapping values favored a suspicion of amyloidosis, more prominent segmental thickening in the mid-inferior and mid-inferoseptal regions remained more characteristic of the HCM group.

Native T1 mapping is a valuable quantitative tool for characterizing myocardial tissue in various diseases presenting with increased wall thickness. While it is well established that native T1 values are characteristically reduced in Fabry disease and markedly increased in cardiac amyloidosis, HCM presents a more complex and heterogeneous phenotypic profile. In HCM, pathological features such as myocyte disarray, interstitial fibrosis, and microvascular dysfunction can lead to varying degrees of elevation in native T1 values [3]. Chronic microvascular ischemia has been shown to contribute to myocardial remodeling and fibrotic changes, which may result in elevated native T1 values, particularly in advanced disease stages [8]. In particular, diffuse fibrosis, advanced-stage disease, and microvascular injury-related T1 elevation in HCM may overlap with the increased T1 values observed in amyloidosis, thereby complicating the differentiation between cardiac amyloidosis and HCM [3]. This overlap underscores the challenge of differentiating between these two conditions solely based on imaging. However, our findings suggest that when interpreted within the appropriate clinical context—and especially when gadolinium-based contrast agents cannot be administered—native T1 mapping provides crucial supportive evidence to raise clinical suspicion for an infiltrative process over a primary cardiomyopathic one.

Although cardiac amyloidosis has been considered a disease in which clinical findings primarily result from ventricular involvement with secondary atrial structural and functional changes, the recent literature suggests that atrial involvement may occur concurrently with ventricular involvement or may develop earlier than ventricular changes [9,10,11]. Cardiac MRI has therefore gained importance for comprehensive tissue characterization beyond conventional morphological assessment. In the present study, atrial size was evaluated; however, atrial tissue characterization could not be performed because contrast administration was not feasible in the majority of patients. In addition, the thin atrial walls limit the reliability of atrial mapping measurements, making optimal tissue characterization technically challenging.

There are several studies in the literature comparing imaging findings of amyloidosis and HCM with both contrast enhanced images and non-contrast images. It is known that contrast enhancement patterns may help differentiate these two diseases. If contrast enhancement is present, subendocardial or transmural LGE patterns are expected in amyloidosis, while LGE is usually seen in right and left ventricle junctions in HCM [2,3]. However, the systemic nature of amyloidosis frequently leads to impaired renal function, which precludes the administration of contrast agents in a significant number of patients. Consequently, establishing reliable, non-contrast-based discriminatory methods has become a clinical necessity. In our study, LGE images were intentionally excluded from the primary analysis to reflect this ‘real-world’ clinical constraint. By focusing on native mapping techniques, we aimed to address the diagnostic gap for patients in whom contrast-enhanced imaging is either contraindicated or unavailable, thereby providing a safer and more widely applicable diagnostic workflow.

Our results are largely consistent with the prospective study by Korthals et al. [12], who evaluated 30 amyloidosis and 20 HCM patients. They reported that myocardial native T1 values were significantly higher in the amyloidosis group compared to both HCM and control groups, achieving a high discriminatory performance (AUC: 0.984). Similarly, our study demonstrated a strong discriminatory value for myocardial T1 (AUC: 0.938). Interestingly, we also found that blood-pool T1 values exhibited high sensitivity and specificity (AUC: 0.900) in differentiating these two conditions. While Korthals et al. did not perform a detailed comparison of myocardial segmental thickness, they noted that thickening in HCM tended to be more asymmetric and prominent in the interventricular septum. Our study extends these findings by providing a segment-by-segment analysis; although the overall presence of myocardial thickening did not differ significantly between the groups, we identified that the mid-inferoseptal and mid-inferior segments were significantly thicker in HCM patients compared to those with suspected amyloidosis. This segmental differentiation may provide additional morphological clues when mapping values are inconclusive.

Consistent with our findings, several other studies have reported significantly higher myocardial native T1 values in amyloidosis compared to HCM [7,8]. Both Steen et al. [1] and Nam et al. [13], utilizing the 5(3)3 MOLLI sequence, observed a similar trend in cohorts of 42 and 46 amyloidosis patients, respectively. In another comparative study involving amyloidosis, HCM, and hypertensive heart disease, a myocardial T1 cut-off value of 1341 ms was identified for differentiating amyloidosis with high diagnostic performance (AUC: 0.994; sensitivity: 100%; specificity: 97%) [14]. In our study, the optimal cut-off value for discriminating between HCM and suspected amyloidosis was 1071 ms (AUC: 0.938). The notable discrepancy in absolute cut-off values between these studies is likely attributable to differences in MRI hardware, and specific vendor-dependent T1 mapping pulse sequences. These variations underscore the critical importance of establishing center-specific reference ranges and cut-off values for native mapping parameters rather than relying on a universal threshold.

Our results are further supported by Fontana et al. [15], who evaluated a large cohort of 138 patients with confirmed systemic amyloidosis (including both AL and ATTR subtypes) and 46 HCM patients. Utilizing the ShMOLLI (Shortened Modified Look-Locker Inversion Recovery) sequence, they reported significantly higher native T1 values in the amyloidosis group (1097 ± 43 ms) compared to HCM patients (1026 ± 64 ms) and healthy controls (967 ± 34 ms). They also noted that T1 elevation was more pronounced in AL amyloidosis than in the ATTR subtype (1130 ± 68 ms vs. 1097 ± 43 ms; *p* = 0.01). Although their absolute T1 values differ slightly from our findings—likely due to the inherent differences between ShMOLLI and the 5(3)3 MOLLI sequence used in our study—the overall trend of significant T1 prolongation in infiltrative disease remains consistent. The ability of native T1 mapping to reflect the increased interstitial space across different subtypes and sequences underscores its robustness as a non-contrast marker for cardiac amyloidosis.

In contrast to our findings, Chatzantonis et al. [16] reported a lower discriminatory performance for native T1 mapping (AUC: 0.76) compared to LGE (AUC: 0.97) in a cohort where MRI findings were correlated with histopathological data from myocardial biopsies. However, they noted that their diagnostic performance analysis for T1 mapping might not be entirely reliable due to the unavailability of mapping data across their entire patient population. Furthermore, their study utilized MRI devices from different vendors and both 1.5 T and 3 T field strengths, which may have introduced significant variability in T1 measurements. In our study, the use of a consistent 1.5 T scanner and a standardized 5(3)3 MOLLI protocol for all subjects likely contributed to the higher and more robust AUC values observed (AUC: 0.938). This consistency underscores the importance of standardized acquisition parameters in achieving high diagnostic performance with non-contrast mapping techniques.

The feasibility of native T1 mapping across different platforms was further highlighted in a multicenter study involving four different MRI scanners from two different vendors. In that study, which included 38 patients with cardiac amyloidosis and 30 healthy controls, researchers utilized various MOLLI sequence schemes (including 3(3)3(3)5 and 5(3)3). Despite the inherent variability introduced by different hardware, pulse sequences, and magnetic field strengths, the study demonstrated that cardiac amyloidosis could be reliably distinguished from healthy controls across all platforms. A key conclusion of that multicenter analysis was the critical necessity for each center to establish its own normal reference T1 values to maintain diagnostic effectiveness [17]. Our study adheres to this principle by utilizing a consistent, single-center protocol, which minimizes inter-scanner variability and reinforces the reliability of our proposed cut-off values.

In our cohort, four patients in the suspected amyloidosis group yielded myocardial T1 values below our calculated cut-off, and two patients showed similar results for blood-pool T1 values. Although these patients exhibited T1 values that were higher than the control group, they did not reach the specific threshold for discriminating amyloidosis from HCM. These cases highlight the existence of a ‘diagnostic grey zone’ where native mapping values alone may be inconclusive. This observation is consistent with the findings of Baggiano et al. [18], who evaluated 441 patients with cardiac amyloidosis and identified a substantial grey zone (1036–1164 ms) in 58% of their population. They suggested that in patients with T1 values falling within this intermediate range—where it is neither low enough to exclude nor high enough to confirm the disease—the administration of contrast material should be strongly reconsidered to achieve a definitive diagnosis. Our findings reinforce this pragmatic approach; while native T1 mapping can potentially identify a significant proportion of patients without the need for contrast, a subset of patients in the ‘grey zone’ will still require a multi-parametric evaluation, including contrast-enhanced MRI or other diagnostic modalities.

Our findings are consistent with a recent meta-analysis suggesting that native T1 mapping provides diagnostic performance comparable to LGE across a wide range of studies [19]. However, beyond confirming this general trend, our study extends current knowledge by adopting a multi-parametric, non-contrast approach that integrates myocardial, blood-pool, and liver T1 mapping with segmental wall thickness analysis. While the literature often focuses on global myocardial values, our data reveal that specific segmental patterns—such as more prominent thickening in the mid-inferoseptal and mid-inferior regions in HCM—can provide critical morphological clues in cases where mapping values are borderline. This combined assessment is particularly paramount for patients with renal impairment, offering a robust and safe diagnostic framework when contrast administration is not feasible.

Beyond prior non-contrast mapping studies that primarily focused on myocardial native T1 values, the present study provides a comprehensive, multi-organ evaluation of myocardial, blood-pool, and liver T1 mapping within the same cohort. Our findings demonstrate that blood-pool T1 mapping provides incremental discriminatory information, while liver T1 mapping appears to have a more limited performance in this specific clinical context. Importantly, this multiparametric, contrast-free approach supports a practical and safe diagnostic workflow for patients in whom gadolinium administration is contraindicated. However, native T1 mapping should not be interpreted as a disease-specific marker but rather as a quantitative parameter reflecting changes in myocardial tissue composition. Given the potential for overlapping T1 values—particularly between advanced-stage HCM and cardiac amyloidosis—our findings underscore the necessity for a cautious interpretation of cut-off values. Our findings regarding liver T1 and T2 mapping should be interpreted as exploratory, as they aim to investigate the systemic nature of amyloid deposition rather than providing a primary diagnostic marker in this specific clinical context. We emphasize that mapping data should always be integrated with clinical history, laboratory biomarkers, and morphological features such as segmental wall thickness and extracardiac findings to achieve the highest diagnostic reliability.

Beyond its diagnostic utility, improved tissue characterization through native T1 and T2 mapping holds significant clinical implications for risk stratification. As synthesized in the recent literature, the precise quantification of myocardial involvement is essential for understanding the substrate of arrhythmic complications, such as atrial fibrillation and sudden cardiac death, which are major contributors to mortality in cardiac amyloidosis [20].

A deliberate decision was made to exclude LGE from the comparative analysis. In our retrospective cohort, the majority of patients with suspected cardiac amyloidosis underwent non-contrast MRI specifically due to impaired renal function. Including LGE data only for the subset of patients who could receive contrast would have introduced significant selection bias and compromised the comparability between the study groups. By focusing exclusively on native parameters, this study intentionally reflects the real-world clinical challenges where contrast-enhanced imaging is frequently contraindicated, thereby establishing the diagnostic value of a purely non-contrast workflow.

Extracellular volume fraction (ECV) mapping shows a strong correlation with amyloid load in patients with amyloidosis, serving as a helpful marker for diagnosis, risk assessment, and clinical follow-up. However, since ECV calculation requires gadolinium administration, our study focused on native mapping parameters to provide a reliable diagnostic alternative for patients with renal contraindications to contrast agents.

The higher prevalence of elevated cardiac biomarkers in the suspected amyloidosis group likely reflects the more advanced clinical stage and systemic nature of the disease at presentation. However, this asymmetry in biomarker availability and distribution between groups may introduce bias and should be considered when interpreting the observed group separation. This limitation reflects the retrospective design and the real-world clinical context in which biomarker data were not uniformly available across all groups.

This study has several limitations that warrant consideration. The primary methodological limitation of this study is the absence of a histological gold standard (endomyocardial biopsy) for the suspected amyloidosis group, which limits a definitive confirmation of the diagnosis. While the lack of systematic biopsy and amyloid subtyping is an important constraint, our cohort reflects real-world clinical practice where invasive procedures are often avoided due to patient frailty. Consequently, our findings represent a differentiation between clinical phenotypes—highly suggestive of infiltrative disease—rather than histologically proven entities. Second, the study’s retrospective, single-center design and relatively small sample size may limit the generalizability of the findings. Confounding factors such as concomitant renal disease or hypertension could not be fully excluded, which may independently influence T1 values. Third, while echocardiographic findings were utilized as supportive clinical criteria during the initial patient selection and group allocation, a direct head-to-head statistical comparison between echocardiographic and MRI-derived functional parameters was not a primary objective of this study. The focus remained on the discriminatory power of non-contrast MRI mapping. Fourth, our study did not involve amyloid subtyping (AL vs. ATTR) or the systematic use of bone scintigraphy. At the time of this retrospective evaluation, bone scintigraphy data were not available within our institutional system for all suspected subjects. While distinguishing between AL and ATTR subtypes is clinically paramount, our primary objective was to evaluate the initial discriminatory power of native MRI mapping in a broad population with suspected cardiac amyloidosis regardless of the specific subtype. Future prospective trials incorporating both multi-tracer bone scintigraphy and native MRI mapping are necessary to determine if specific T1 or T2 thresholds can reliably differentiate between these amyloid subtypes without the need for contrast or biopsy. Furthermore, a significant age disparity existed between the control group and the patient cohorts. While this reflects typical referral patterns—where normal MRI findings are more common in younger individuals—and despite evidence suggesting that age-related T1 changes are generally modest [21], this remains a limitation for interpreting absolute values. Additionally, T1 and T2 measurements were obtained via manually defined ROIs rather than automated segmental bull’s-eye mapping, potentially introducing operator-dependent variability. To mitigate this, a consistent methodology was applied across all subjects. Crucially, the omission of LGE imaging, necessitated by the high prevalence of renal impairment in our cohort, remains a significant methodological constraint. While this reflects clinical reality, it precludes the definitive tissue characterization typically provided by contrast enhancement patterns and increases the reliance on native mapping values. Lastly, we acknowledge that the one-year biomarker inclusion window is a potential source of variability and clinical inconsistency. Although most measurements were performed within a narrow temporal window, the progressive nature of infiltrative disease means that wider intervals could influence thecorrelations between biochemical and imaging findings.. Finally, the well-known dependence of mapping values on scanner hardware and sequence parameters necessitates caution when applying our specific cut-off values to other centers. The absence of an external validation cohort and inter-vendor comparisons further limits the immediate clinical translation of our results. Future larger, multicenter prospective trials are warranted to validate these findings across broader populations and standardized protocols.

Native T1 and T2 mapping should not be viewed as a direct replacement for contrast-enhanced MRI or echocardiography, but rather as a complementary diagnostic layer. It is particularly valuable in specific clinical scenarios, such as patients with unexplained myocardial hypertrophy where gadolinium-based contrast agents are contraindicated. In these instances, native mapping provides critical tissue characterization that extends beyond conventional morphological assessment. However, while our study demonstrates strong discriminatory performance, these findings should be interpreted within a multimodality diagnostic framework, ensuring that quantitative mapping values are always correlated with clinical and laboratory data. Furthermore, since native T1 values may not show significant elevation in subclinical stages or early phenotypic expressions, relying on a single mapping modality may lead to diagnostic overlap. Our results underscore the importance of a composite assessment to enhance diagnostic reliability in complex cardiomyopathies.

## 5. Conclusions

In conclusion, native T1 mapping appears to be a potentially valuable non-contrast tool for differentiating suspected amyloidosis from hypertrophic cardiomyopathy. Our findings suggest that integrating myocardial and blood-pool T1 values with segmental morphological assessment may provide supportive diagnostic information. This multiparametric, contrast-free approach could offer a practical alternative for the clinical evaluation of patients in whom gadolinium administration is contraindicated or deferred. However, given the retrospective nature of this study, the limited sample size, and the lack of external validation, these results should be interpreted with caution. Specifically, the identified thresholds should be viewed as center-specific and illustrative of the marked tissue characterization differences between phenotypes, rather than universal diagnostic cut-offs. Further prospective, multicenter studies are warranted to confirm these thresholds and to evaluate their clinical utility across broader patient populations.

## Figures and Tables

**Table 1:** Comparison of T1 and T2 values among the control, amyloidosis, and HCM groups.

	Control (*n* = 20)	Amyloidosis (*n* = 20)	HCM (*n* = 20)	*p* *	*p* **	*p* ***
T1 myocardium (ms)	1005 ± 22	1114 ± 59	1022 ± 50	<0.001	0.183	<0.001
T1 blood-pool (ms)	1573 ± 96	1726 ± 122	1511 ± 114	<0.001	0.071	<0.001
T1 liver (ms)	591 ± 56	650 ± 85	588 ± 68	<0.001	0.855	0.014
T2 myocardium (ms)	46 ± 3	50 ± 3	48 ± 3	<0.001	0.094	0.005
T2 blood-pool (ms)	167 ± 26	182 ± 36	170 ± 28	0.147	0.724	0.256
T2 liver (ms)	50 ± 5	48 ± 6	50 ± 4	0.199	0.694	0.271

*p* *: Control versus Amyloidosis. *p* **: Control versus HCM. *p* ***: Amyloidosis versus HCM.

**Table 2:** Diagnostic performance of T1 and T2 values for differentiating amyloidosis from controls.

	Cut-Off Value	AUC	Sensitivity	Specificity	PPV	NPV	Accuracy
T1 myocardium	1061	0.975 (<0.001)	0.85 (0.64–0.95)	1 (0.84–1)	1 (0.89–0.99)	0.87 (0.72–0.95)	0.925
T1 blood-pool	1627.5	0.836 (<0.001)	0.85 (0.64–0.95)	0.65 (0.43–0.82)	0.71 (0.54–0.84)	0.81 (0.65–0.91)	0.75
T1 liver	622	0.710 (0.23)	0.60 (0.39–0.78)	0.65 (0.43–0.82)	0.63 (0.47–0.77)	0.62 (0.45–0.76)	0.625
T2 myocardium	48.5	0.816 (0.001)	0.95 (0.70–0.97)	0.80 (0.58–0.92)	0.82 (0.66–0.92)	0.89 (0.74–0.96)	0.850

AUC: Area under the curve. PPV: Positive predictive value. NPV: Negative predictive value.

**Table 3:** Diagnostic performance of T1 and T2 values for differentiating amyloidosis from HCM.

	Cut-Off Value	AUC	Sensitivity	Specificity	PPV	NPV	Accuracy
T1 myocardium	1071	0.938 (<0.001)	0.80 (0.58–0.91)	0.85 (0.64–0.95)	0.84 (0.68–0.93)	0.81 (0.65–0.91)	0.825
T1 blood-pool	1597	0.900 (<0.001)	0.90 (0.70–0.97)	0.90 (0.70–0.97)	0.90 (0.75–0.97)	0.90 (0.75–0.97)	0.900
T1 liver	598	0.724 (0.015)	0.70 (0.48–0.85)	0.60 (0.39–0.78)	0.64 (0.47–0.78)	0.67 (0.50–0.80)	0.650
T2 myocardium	49.5	0.756 (0.006)	0.80 (0.58–0.92)	0.75 (0.53–0.89)	0.76 (0.60–0.88)	0.79 (0.63–0.90)	0.775

AUC: Area under the curve. PPV: Positive predictive value. NPV: Negative predictive value.

**Table 4:** Comparison of EF, EDVi, and atrial enlargement between amyloidosis and HCM groups.

	Amyloidosis	HCM	*p*
EF (%)	55 ± 12	63 ± 11	0.021
EDVi (mL/m^2^)	77 ± 30	69.6 ± 20	0.509
Atrium enlargement	*n* = 11 (55%)	*n* = 8 (40%)	0.080

EF: Ejection fraction; EDVi: End-diastolic volume index.

## Data Availability

The data presented in this study are available from the corresponding author upon reasonable request. The data are not publicly available due to patient privacy and ethical restrictions.

## Abbreviations

The following abbreviations are used in this manuscript:

AUC	Area Under the Curve
EDVi	End-Diastolic Volume Index
EF	Ejection Fraction
FOV	Field of View
GRAPPA	Generalized Autocalibrating Partial Parallel Acquisition
HCM	Hypertrophic Cardiomyopathy
LGE	Late Gadolinium Enhancement
MOLLI	Modified Look-Locker Inversion Recovery
MRI	Magnetic Resonance Imaging
NPV	Negative Predictive Value
PPV	Positive Predictive Value
ROC	Receiver Operating Characteristic
ROI	Region of Interest
SSFP	Steady-State Free Precession
TE	Echo Time
TR	Repetition Time
